# Altered cytokine profiles of human retinal pigment epithelium: Oxidant injury and replicative senescence

**Published:** 2013-03-21

**Authors:** Sijia Cao, Gregory B. Walker, Xuefeng Wang, Jing Z. Cui, Joanne A. Matsubara

**Affiliations:** Department of Ophthalmology and Visual Sciences, University of British Columbia, Vancouver, British Columbia, Canada

## Abstract

**Purpose:**

Age-related macular degeneration (AMD) is a local, chronic inflammatory disease of the eye that is influenced by oxidative stress and dysregulation of the retinal pigment epithelium (RPE) associated with aging. The purpose of this study is to characterize the effects of oxidative stress and replicative senescence on the secreted cytokine profiles of RPE in vitro.

**Methods:**

We used multiple, serial passages of human RPE cells from primary culture as an in vitro model of aging. Responses of early passage 5 (P5) and late passage 21 (P21) RPE cells were compared. Oxidative stress was induced in RPE cells (P5) by exposure to 75 μM hydroquinone (HQ) for 24 h. The secretome profiles of the RPE cells were measured with a multiplex suspension assay that assayed human cytokine, chemokine, and growth factors. Immunohistochemistry on younger (≤55 years old) and older (≥70 years old) human post-mortem donor eyes was used to verify selected cytokines.

**Results:**

Supernatant of HQ-treated RPE cultures exhibited increased secreted levels of vascular endothelial growth factor (VEGF), interleukin (IL)-12, and IL-10 that reached statistical significance (p<0.05). Supernatant of late passage P21 RPE cultures exhibited decreased secreted levels of stromal cell-derived factor (SDF)-1α, granulocyte macrophage colony-stimulating factor (GM-CSF), IL-8, IL-15, IL-6, and an increased level of IL-1ra compared to early passage P5 RPE cultures that reached statistical significance (p<0.05). Immunohistochemical analysis demonstrated increased expression of IL-1ra in RPE cells from older post-mortem donor eyes (≥70 years old) versus younger eyes (≤55 years old).

**Conclusions:**

Our data demonstrate a unique cytokine secretion profile of primary culture RPE cells at early and late passage. Our in vitro data suggest an age-specific modulation of cytokine secretion in RPE and is consistent with immunohistochemical analysis on post-mortem eyes. The secretion profile associated with RPE under conditions that mimic oxidative stress, another factor associated with the pathogenesis of AMD, emphasizes upregulation of the angiogenic growth factor, vascular endothelial growth factor. Together, these data support the role of advanced age and oxidative stress in inflammatory cytokine modulation in RPE cells.

## Introduction

Age-related macular degeneration (AMD) is a multifactorial degenerative disease characterized by retinal cell atrophy and/or choroidal neovascularization in the macula. The disease constitutes the number one cause of blindness among the elderly in industrialized countries [1]. The management of the exudative form AMD featured by choroidal neovascularization has been greatly improved due to the development of anti-vascular endothelial growth factor (VEGF) therapies [2]. However, the exudative form of AMD accounts for only approximately 10% of the total AMD cases [2,3]. In other words, effective measures are still lacking to cure or prevent the progression of about 90% of AMD cases due to insufficient knowledge of the underlying mechanisms.

Pigmentary abnormalities and morphological changes in retinal pigment epithelium (RPE) are associated with sub-RPE drusen and advanced AMD lesions, which suggest that RPE cells might play an important role in AMD pathogenesis [4,5]. Indeed, the high metabolic rate and multiple physiologic functions of the RPE subject these cells to cumulative oxidative stress [6]. With aging, RPE cells not only decrease in numbers, but also lose melanin and accumulate lipofuscin, leading to a decreased antioxidant capacity [7-10]. These age-related changes in RPE may explain why aging and oxidative stress constitute important risk factors for AMD. However, the specific molecular and cellular pathway(s) associated with aging and oxidative stress and the triggered downstream events associated with the pathogenesis of AMD are still unclear but may involve inflammation [11].

In this study, we focus on the secreted levels of inflammatory cytokines and growth factors after multiple passage of human RPE cells in culture. Multiple passage of cells in culture was shown to lead to a cellular state of replicative senescence, which is widely used as an in vitro model of aging [12,13]. Earlier studies demonstrated that late passage (P21) RPE cells from primary culture demonstrated a reduced capacity for cellular division as RPE cells from aged donors. In addition, these cells have many other aging characteristics such as senescence-associated β-galactosidase buildup and telomere loss [14,15]. Therefore, P21 RPE cells from human primary culture can be used to study senescence in RPE cells. Earlier work by other laboratories showed that the altered gene expression profiles in late passage RPE cells were mostly related to stress and matrix regulation [16,17]. Our earlier work was one of the first to report that genes related to inflammation were also altered in this in vitro model [13]. Given that chronic inflammation in the outer retina appears to be an early trigger for AMD [4,18], the present study focused on evaluating the inflammatory gene changes at the protein level to understand the changes in RPE behavior associated with replicative senescence and oxidative stress in vitro.

To study the effect of oxidative stress, another risk factor for AMD, we exposed RPE cells to hydroquinone (HQ). HQ induces oxidative stress in several cell culture systems, including RPE [19-21]. Previous work showed that HQ induced changes in gene expression associated with multiple cellular functions such as extracellular matrix turnover, apoptosis, and angiogenesis, but whether proinflammatory regulators are also affected by HQ-induced oxidative stress is unknown. Therefore, we hypothesize that the two factors associated with the pathogenesis of AMD, replicative senescence (an in vitro model of aging) and oxidative stress of RPE will promote proinflammatory pathways.

## Methods

### Cell culture of primary human retinal pigment epithelial cells

Human RPE cells used in all experiments described here were isolated from fetal donor eye tissues as described previously [13,22]. Methods for securing human tissue were humane and included proper written informed consent, which complied with the Declaration of Helsinki. Human fetal donor eyes had no known pathology and were used under the guidelines and regulation of the Research Ethics Board at the University of British Columbia, Vancouver, Canada. Briefly, after the vitreous and the neural retina were removed, the eyecups were washed and incubated in Eagle’s minimum essential medium (MEM, Life Technologies, Burlington, ON) with 0.05% trypsin (Fisher Scientific, Ottawa, ON) and 0.02% EDTA (Fisher Scientific) for 1 h. This was followed by the release of RPE cells by gentle pipetting. The collected cells were washed two times and plated in laminin-coated flasks. The RPE cells were maintained in a humidified incubator of 5% CO_2_ and 95% air. The MEM medium was supplemented with 10% fetal bovine serum (FBS; Fisher Scientific).

Late passage RPE cells were obtained by serial passage and were characterized by increased histochemical staining of β-galactosidase and telomere shortening as described before [13,15,16]. Confluent cells were repeatedly trypsinized and subcultured at a 1:4 ratio, until the required passage number was reached and senescent characteristics were observed (data not shown). Population doublings for each passage were estimated by assuming two population doublings per passage as described earlier [13]. Supernatant from late passage (P21) and early passage (P5) cells, harvested at 24 h after culture in 1% FBS-containing MEM, were used in this study. The early passage cells were polygonal, while the late passage cells were flattened and fusiform in morphology (data not shown).

For HQ stimulation studies, human RPE cells at P5 were treated with HQ at the concentration of 50 μM, 100 μM, 200 μM, 300 μM, and 400 μM for 24 h or 48 h in Dulbecco's Modified Eagle Medium (DMEM, Fisher Scientific) containing 1% FBS, respectively. P5 RPE cells cultured in 1% FBS-DMEM without HQ were used as an untreated positive control for the cell viability assay.

### Cell viability assay

RPE cell viability was measured with methylthiazolyldiphenyl-tetrazolium bromide (MTT) assay and/or lactate dehydrogenase (LDH) assay. For MTT assay, 50 µl of MTT solution (2 mg/ml; Sigma-Aldrich, Oakville, ON, Canada) was added to each well of RPE cells treated by a series of gradient concentration of HQ, and the wells were incubated at 37 °C for 4 h. Mitochondrial and cytosolic dehydrogenases of living cells reduced the yellow tetrazolium salt (MTT) to a purple formazan dye that was then detected with spectrophotometry. After incubation, the MTT solution was aspirated, and 150 µl of DMSO was added for a period of 20 min. Optical densities of the supernatant were read at 550 nm using a microplate spectrophotometer (Bio-Tek Instruments, Inc., Winooski, VT). Absorbance was normalized to the untreated positive control cultures, which represented 100% viability. Two independent experiments in quadruplicate were performed in this study.

For the LDH assay, RPE cell concentrations from different culture conditions were optimized, and the supernatants were collected. The LDH concentration within the supernatants was measured with an LDH kit (Clontech Laboratories, Mountain View, CA). The kit coupled a multistep LDH-catalyzed conversion of lactate to pyruvate to reduction of tetrazolium (yellow) to formazan (red). A rise in the LDH due to membrane damage following cell death correlates to the amount of red formazan produced, which was detected with spectrophotometry (absorbance at 490 nm). Every measurement was done in triplicate.

### Bio-Plex cytokine assays

The secretion profiles of RPE cells under analysis were measured with Bio-Plex Pro human cytokine, chemokine, and growth factor assays as described by the manufacturer (Bio-Rad Laboratories, Hercules, CA). Three independent experiments in duplicate were performed for each condition. The assays use xMAP technology, which permits the quantification of multiple cytokines in a single well with 50 μl of sample. In our experiments, the premixed multiplex beads of the Bio-Plex human cytokine 27-plex assay (Bio-Rad Laboratories) and an additional 3-plex were used. They included the following cytokines/growth factors: Interleukin (IL)-1β, IL-1ra, IL-2, IL-4, IL-5, IL-6, IL-7, IL-8, IL-9, IL-10, IL-12 (p70), IL-13, IL-15, IL-17, basic fibroblast growth factor (FGF basic), eotaxin, granulocyte colony-stimulating factor (G-CSF), granulocyte macrophage colony-stimulating factor (GM-CSF), interferon-gamma (IFN-γ), interferon gamma-induced protein 10 (IP-10), monocyte chemoattractant protein (MCP)-1, MCP-3, macrophage inflammatory protein (MIP)-1α, MIP-1β, platelet-derived growth factor (PDGF)-BB, regulated and normal T cell expressed and secreted (RANTES), stromal cell-derived factor (SDF)-1α, tumor necrosis factor (TNF)-α, vascular endothelial growth factor (VEGF), TNF-related apoptosis-inducing ligand (TRAIL).

Briefly, 50 μl of cytokine standards and samples (culture supernatants from HQ-treated cells and untreated cells, early passage cells and late passage cells) were incubated with 25 μl of anticytokine conjugated beads in 96-well filter plates for 30 min at room temperature, with agitation (1,100 rpm for 30 s and then 300 rpm for 30 min). After incubation, plates were washed three times via vacuum filtration with 100 μl of Bio-Plex wash buffer per well using the Bio-Plex Pro wash station. Plates were then incubated with 25 μl of diluted biotinylated detection antibody for 30 min at room temperature with agitation. After another three washes, 25 μl of streptavidin–phycoerythrin were added in each well, and the plates were incubated for 10 min at room temperature with agitation. Following another three washes, the beads were resuspended in 125 μl of Bio-Plex assay buffer, and vortexed for 30 s at 1,100 rpm and further incubated for 2 min at 300 rpm. Standards and samples were analyzed using the Bio-Plex 200 Suspension Array System, and subsequent raw median fluorescent intensity data were captured and analyzed using Bio-Plex Manager software 4.1 (Bio-Rad Laboratories).

### Immunohistochemistry

Human eyes were obtained from the Eye Bank of British Columbia (Vancouver, BC, Canada). Methods for securing human tissue were in compliance with the Declaration of Helsinki. The protocol was approved by the Clinical Research Ethics Board (CREB) at the University of British Columbia. All tissue samples included in this study were considered normal and excluded tissues from donors with any of the following: evidence of systemic or local infection, progressive central nervous system disease or systemic disease of unknown etiology, lymphoproliferative or myeloproliferative disorders, intrinsic eye disease, or previous ocular surgery. Eye tissues were 10% formalin-fixed and embedded in paraffin to obtain 6-µm sections through the pupil and optic nerve axis. Paraffin sections were deparaffinized and rehydrated by standard procedures. After antigen retrieval in proteinase K (20 μg/ml) for 30 min at room temperature, sections were blocked with 0.3% H_2_O_2_ for 15 min and 5% goat or rabbit serum with 0.3% Triton X-100 for 45 min. Antibody against IL-1ra (1:300, Santa Cruz Biotechnology, Santa Cruz, CA) was applied as primary antibodies at 4 °C overnight. Primary antibody omission or nonimmune isotype antibody was used as negative controls. Sections were then incubated in appropriate secondary antibodies and were developed with Elite® ABC standard kit followed by Vector® VIP Substrate kit (Vector Laboratories, Burlingame, CA).

### Statistical analysis

Results are expressed as mean±standard deviation (SD) or standard error of the mean (SEM). The Student *t* test was used to determine differences between groups. p<0.05 was considered statistically significant.

## Results

### Retinal pigment epithelium cell viability after exposure to hydroquinone

**Figure 1:**
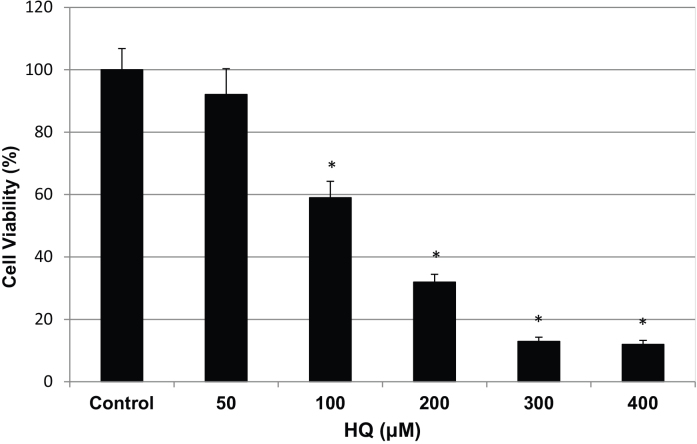
Cell survival of human RPE cells from primary culture exposed to HQ for 24 h. Primary RPE cells (P5) were exposed to 50, 100, 200, 300, and 400 µM HQ in 1% FBS-DMEM for 24 h. The RPE cell survival rate displayed a dose-dependent pattern and resulted in 92%, 59%, 32%, 13%, and 12% cell survival rates with the increase of HQ concentration in medium (mean±SD). Those cultured in 1% FBS-DMEM without HQ served as a positive control, which represented 100% cell survival. P value was obtained by comparing with positive control (Asterisks: p<0.05).

To determine the appropriate concentration of HQ and duration of stimulation, we conducted a dose response study using several concentrations of HQ exposure. We used a MTT assay to examine the effects of HQ stimulation on the viability of primary human RPE cell culture (P5) after 24 h. RPE cells displayed a dose-dependent pattern in survival rate. Exposure to HQ at concentrations from 50 μM to 400 μM for 24 h resulted in 92%, 59%, 32%, 13%, and 12% cell survival compared to RPE cells without HQ exposure (Figure 1). The decrease in the survival rate was significant when RPE cells were treated with 100 μM, 200 μM, 300 μM, and 400 μM, but not with 50 μM HQ (p<0.05). For comparability with our previous studies on RPE cells challenged with drusen components in early AMD-like lesions, we chose a dosage of 75 μM for 24 h to induce a similar RPE survival rate of approximately 85%.

**Figure 2:**
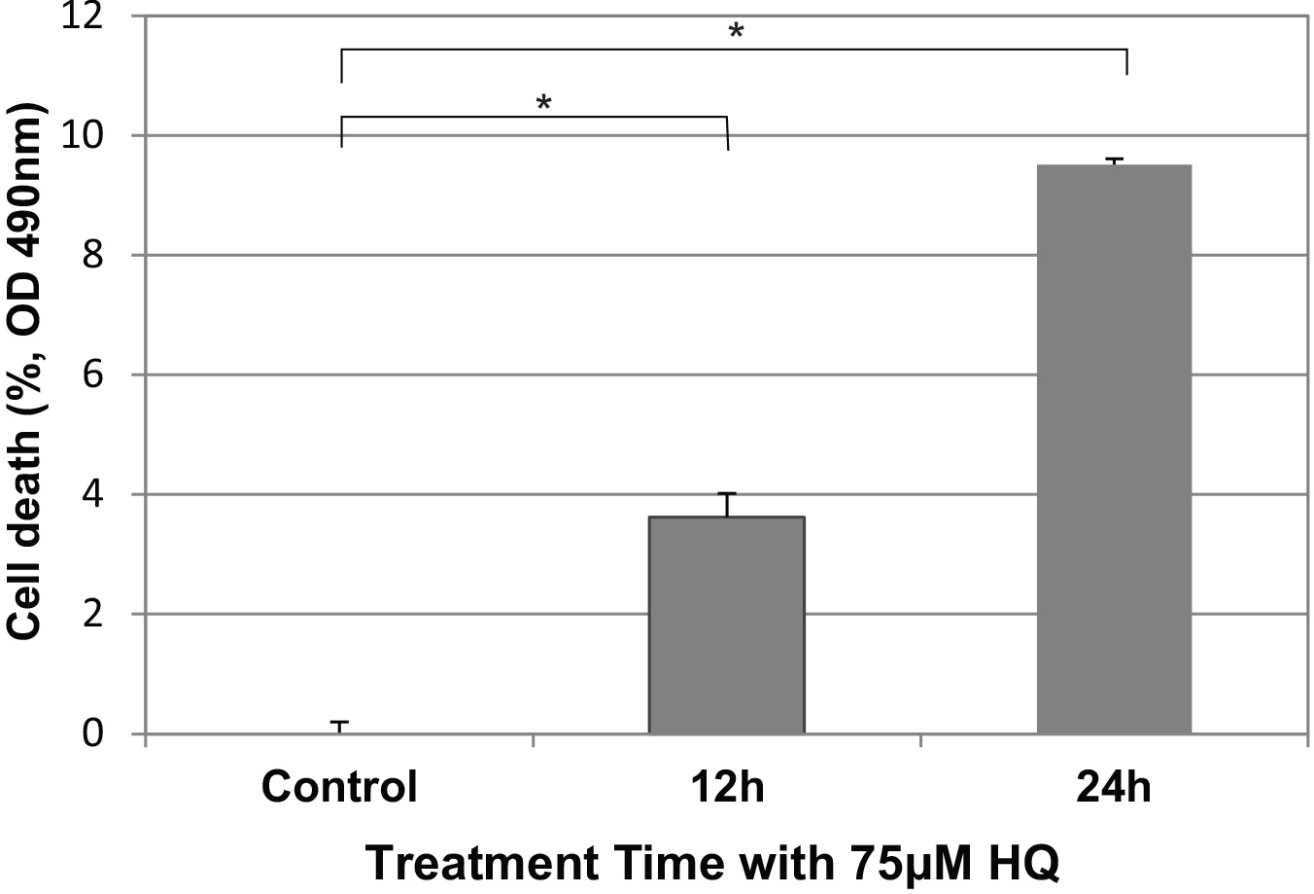
Cell death of human RPE cells from primary culture exposed to 75 μM HQ. LDH assays demonstrated the time-dependent cytotoxicity of 75 μM HQ on primary RPE culture (P5). The 3.6% and 9.5% cell death rates occurred at 12 h and 24 h after exposure to 75 µM HQ, respectively (mean±SD). RPE culture at the starting point of exposure served as control, which represented 0% cell death. The HQ-induced cell death reached a significance level of p<0.05 at 12 h or 24 h compared with control. p<0.05 (shown with asterisks).

When the RPE cells were stimulated with 75 μM HQ, the LDH assays showed that 9.5% of the RPE cells died at 24 h and 3.6% at 12 h post-stimulation (Figure 2). The differences compared to RPE without HQ exposure were significant (p<0.05).

### Cell viability comparison of early passage (P5) and late passage (P21) retinal pigment epithelial cells

**Figure 3:**
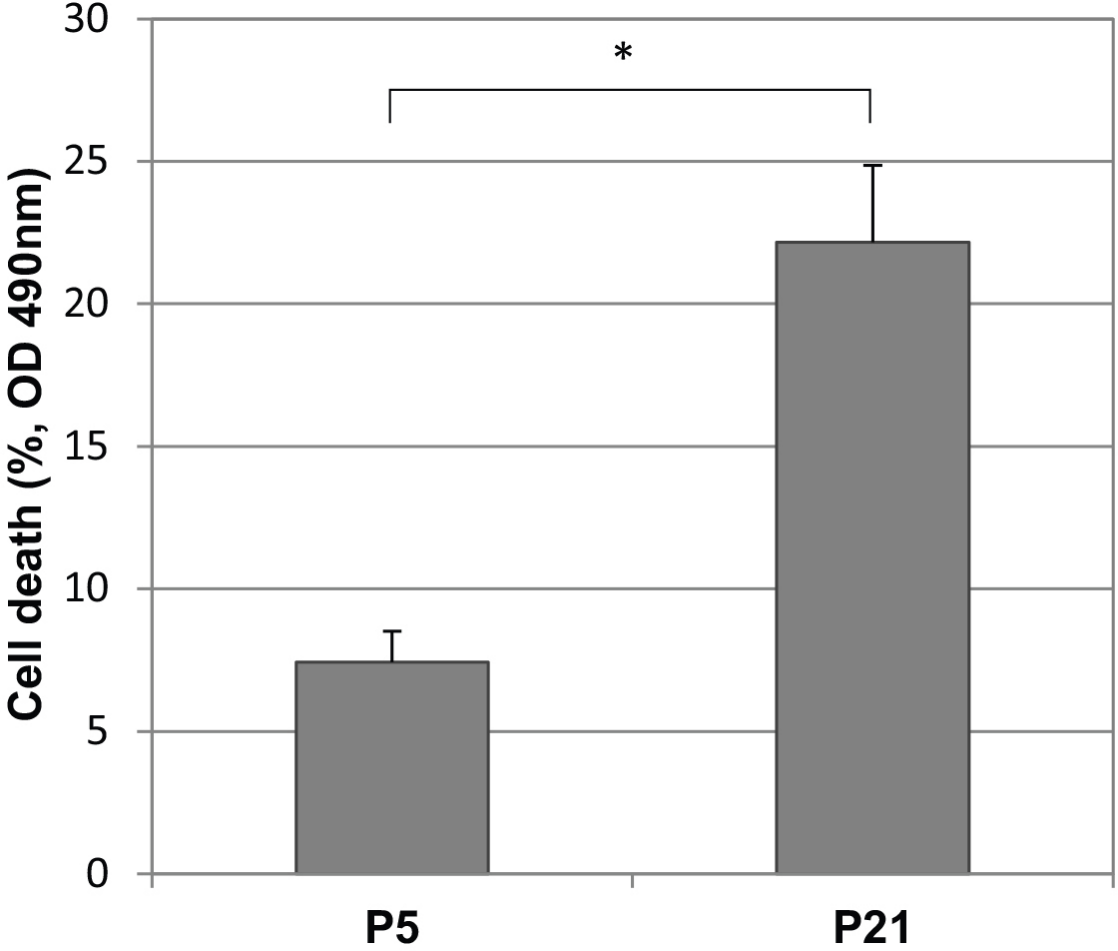
Spontaneous cell death of human RPE cells from primary culture at early (P5) and late passage (P21). LDH assays demonstrated the passage-dependent spontaneous cell death of RPE cells. Cell death rates at P21 and P5 were 22.2% and 7.4%, respectively (mean±SD). The maximum LDH activity released from the cells at the same passage in response to Triton X-100 represented 100% cell death correspondingly. The difference between P5 and P21 was statistically significant (p<0.05, shown with asterisks).

As expected, in an LDH assay, the high passage (P21) RPE cells demonstrated a greater spontaneous cell death rate of approximately 22.2%. Early passage (P5) cells demonstrated 7.4% cell death, and the difference was significant (p<0.05; Figure 3). Thus, the spontaneous death rate was higher in the P21 RPE cells compared with the P5 RPE cells.

### Secretion profile of retinal pigment epithelial cells challenged with 75 μM hydroquinone for 24 h

**Figure 4:**
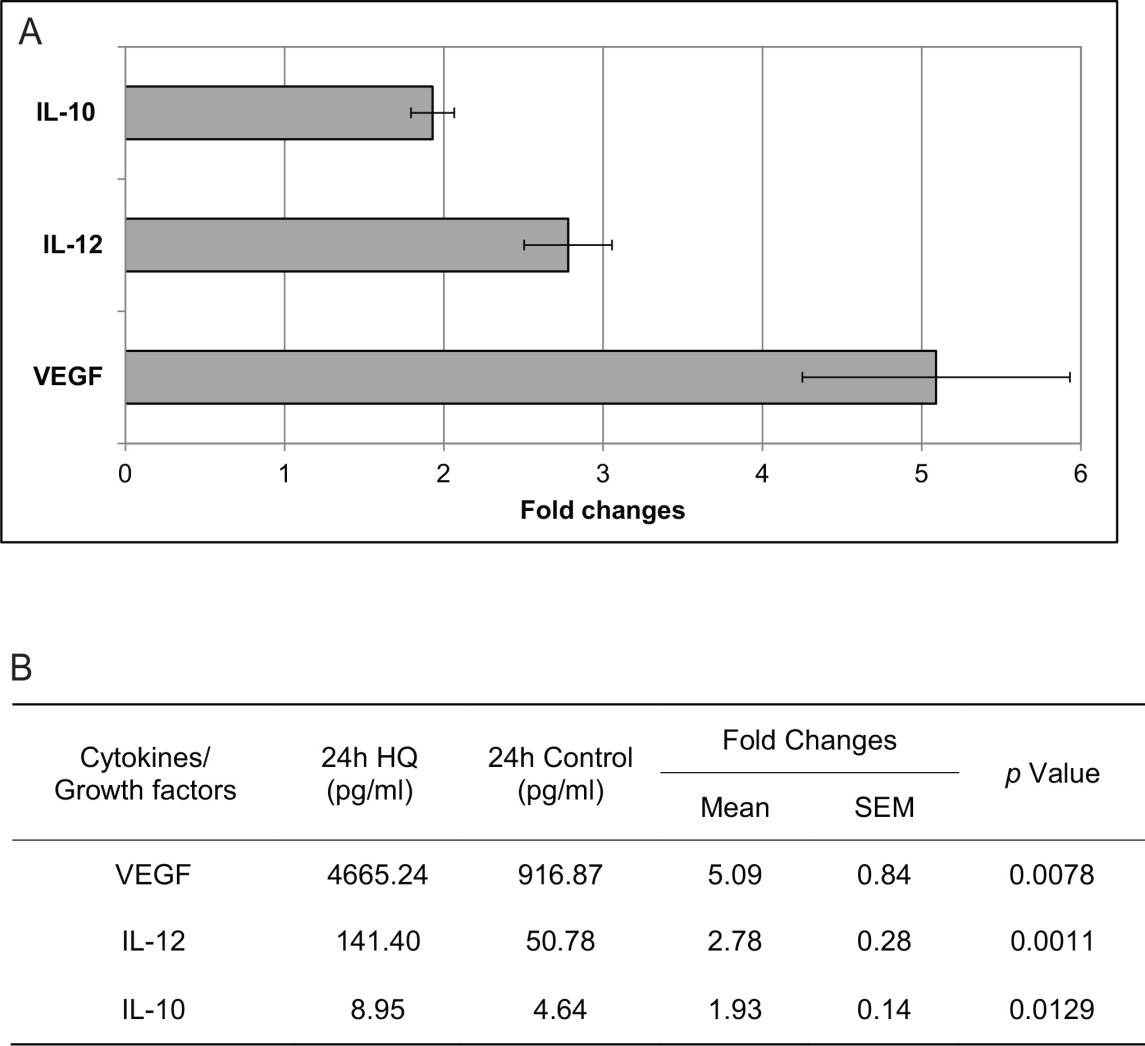
The effect of hydroquinone (HQ) on retinal pigment epithelium (RPE) cytokine and growth factor secretion. **A**: Secreted cytokine/growth factor levels in the culture supernatant of RPE exposed to 75 μM HQ for 24 h were expressed as fold changes over unexposed controls (mean±SEM). When compared to control samples, three cytokines/growth factors were overexpressed by more than 1.5 times and reached a significance level of p<0.05: VEGF, IL-12, and IL-10. **B**: The changes in cytokines/growth factor secretion were compared between from RPE cells exposed to 75 μM HQ for 24 hours and controls.

To evaluate the secreted cytokines/growth factors by RPE after exposure to HQ, we used a suspension array system (Bio-Rad Laboratories). Of the 30 cytokines/growth factors tested, only three, VEGF, IL-12, and IL-10, were overexpressed and reached a significance level of p<0.05 and at a threshold of >1.5-fold difference (Figure 4). The differential expression of the other cytokines tested did not reach significance (Appendix 1).

### Secretion profile of late passage retinal pigment epithelial cells

**Figure 5:**
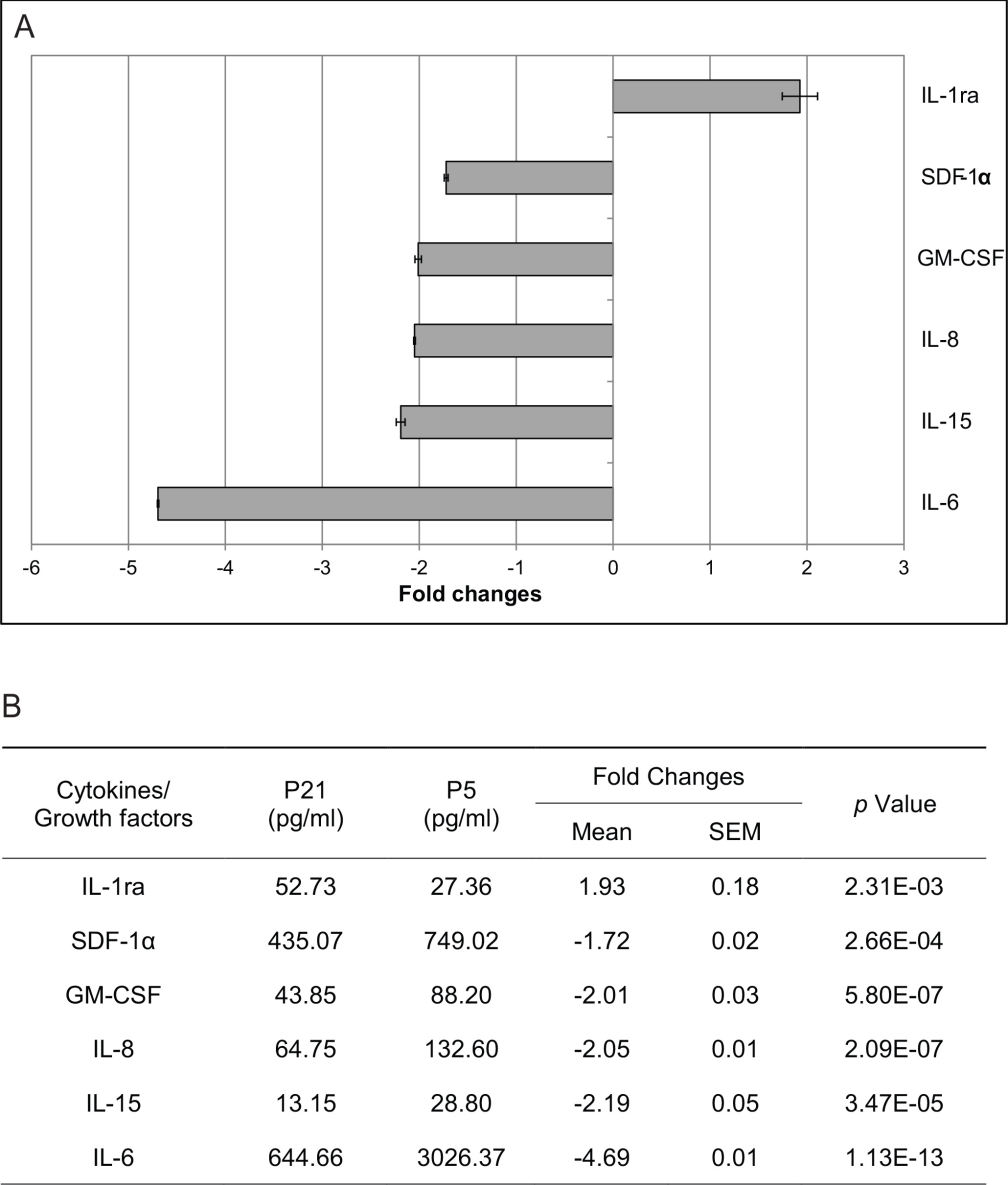
The effect of passage-related senescence on retinal pigment epithelium (RPE) cytokine and growth factor secretion. **A**: Secreted cytokine levels were expressed as fold changes (mean±SEM): Those predominantly in late passage cultures were given positive values; those predominantly in early passage cultures were given negative values. IL-1ra was overexpressed, while SDF-1α, GM-CSF, IL-8, IL-15, and IL-6 were downregulated in late passage (P21) RPE cells compared to early passage (P5) RPE cells. The changes reached a significance level of p<0.05 and a cut-off fold of 1.5. **B**: The changes in cytokine and growth factor secretion were compared between late passage (P21) and early passage (P5) RPE.

We then examined the secretion profile of RPE cells at early and late passage. The results showed that five cytokines/growth factors were differentially downregulated in late passage (P21) RPE cells: SDF-1α, GM-CSF, IL-8, IL-15, and IL-6, while IL-1ra was differentially overexpressed compared to early passage (P5) RPE cells. These changes reached a significance level of p<0.05 and at a threshold of >1.5-fold difference (Figure 5). The differential expression of the other cytokines tested did not reach significance (Appendix 2).

### Interleukin-1ra expression in retinal pigment epithelial cells from human post-mortem donor eyes

**Figure 6:**
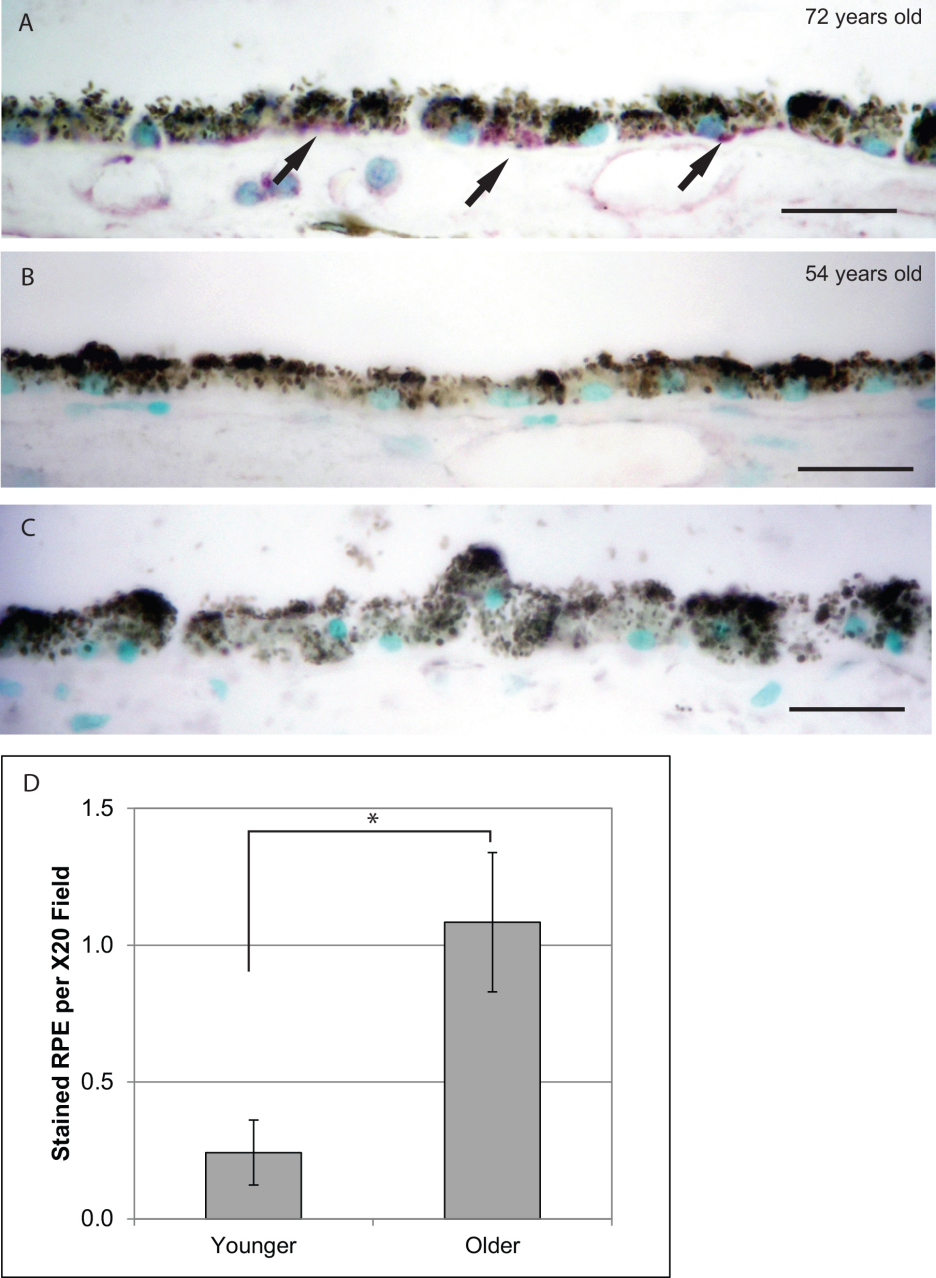
IL-1ra immunoreactivity in RPE of older versus younger human donor eyes. Immunoreactivity for IL-1ra was developed with VIP (purple) and counterstained with methyl green (green; scale bar: 20 μm). **A**: Representative picture of IL-1ra in RPE from a 72-year-old donor eye. **B**: Representative picture of IL-1ra in RPE from a 54-year-old donor eye. **C**: Negative control by primary antibody omission. **D**: IL-1ra was more expressed in RPE from older donor eyes (≥70 years old, n=12) compared with that from younger donor eyes (≤55 years old, n=11). (mean±SEM, *: p<0.01)

Next, we performed immunohistochemistry for IL-1ra in human post-mortem donor eyes and compared the expression level in the RPE layer between older donors (≥70 years old; range 70–80 years, mean age 73.25 years) and younger donors (≤55 years old; range 28–55 years, mean age 47.55 years; Table 1). We found that IL-1ra immunoreactivity was stronger in RPE cells from older donor eyes (≥70 years old) compared with those from younger donor eyes (≤55 years old; Figure 6). This result is consistent with the in vitro data showing that senescent P21 cells overexpress IL-1ra compared to P5 cells.

**Table 1:** Characteristics of human subjects

**Subject**	**Age**	**Gender**	**Diagnosis**
**Older Group (≥70 years old)**			
**1**	72	M	Respiratory arrest
**2**	74	M	Bowel cancer
**3**	73	M	Liver and lung cancer
**4**	73	M	Pulmonary fibrosis
**5**	80	M	Acute myocardial infarction
**6**	72	M	Renal cancer
**7**	72	F	Gastroenteritis and chronic renal failure
**8**	75	M	Cardiac arrest
**9**	74	M	Bone cancer with metastasis
**10**	72	M	Pancreatic cancer
**11**	70	M	Colon cancer with metastasis
**12**	72	F	Cerebrovascular accident, large left hemisphere
**Younger Group (≤55 years old)**			
**13**	47	F	Lung cancer with metastasis
**14**	48	F	Liver cancer, cirrhosis
**15**	55	M	Metastatic esophageal cancer
**16**	44	M	Brain cancer
**17**	55	F	Lung cancer
**18**	54	F	Glioblastoma
**19**	55	F	Lung cancer
**20**	55	M	Prostate cancer
**21**	47	M	Appendix cancer
**22**	28	F	Ovarian cancer with metastasis
**23**	35	F	Cervical cancer with metastasis

## Discussion

The major risk factors for AMD are aging, accumulated oxidative stress, and drusen [23,24]. Our earlier work focused on the effects of drusen components, such as advanced glycation end products and amyloid beta on RPE gene expression in vitro [22,25]. These earlier studies revealed changes at the level of the transcriptome associated with primarily an inflammatory response induced by drusen components. Because little is known about the interactions and differential expression of inflammatory mediators associated with oxidative stress and aging, in this study we focused on the effects of HQ (for oxidative stress) and multiple passage number (for aging) on the secretion profiles of human RPE cells. We hypothesized that under oxidative stress and the aging process, proinflammatory cytokine secretion by RPE is upregulated and thus may further contribute to AMD progression. Combined with our earlier stimulation studies in which we used advanced glycation end products and amyloid beta, two known components of drusen, these data provide information on the RPE inflammatory response in vitro to multiple risk factors of AMD.

### Effects of hydroquinone stimulation

As the effect of HQ stimulation is dose dependent, we chose an HQ concentration that resulted in approximately 85% cell survival (15% cell death) at 24 h to be consistent with our earlier stimulation studies [25]. When exposed to 75 μM HQ for 24 h, VEGF, IL-12, and IL-10 were all significantly oversecreted by the RPE cells (Figure 4). VEGF had the highest fold changes among the growth factors and cytokines examined (fold change=5.09), consistent with other studies [21,26]. VEGF may serve as a survival factor for several cell types in the retina, including RPE and endothelial cells [27]. Byeon et al. found that neutralization of VEGF resulted in RPE death under H_2_O_2_-induced oxidative stress [28]. VEGF is also an angiogenic factor and plays important roles in many ocular neovascular disorders [29]. The upregulation of VEGF induced by HQ exposure might indicate the predisposition of the microenvironment to angiogenesis by induced oxidative injury.

We also observed the overexpression of two cytokines: IL-12 and IL-10. The main function of IL-12 is to induce IFN-γ production from natural killer and T cells, enhancing cell-mediated immunity [30]. With T lymphocytes present in AMD lesions [31], IL-12 may execute other downstream effects such as antiangiogenesis through IFN-γ to balance the angiogenic effect of VEGF [32]. In contrast, IL-10 possesses anti-inflammatory properties and can dampen the production of proinflammatory mediators from immune cells [33,34]. IL-10 may also inhibit IL-12 production, which may compromise the extent of inflammation in the microenvironment [30]. The simultaneous overexpression of cytokines of opposite functions suggests a parainflammatory state of the RPE cells in which they maintain homeostasis under HQ-induced oxidative injury. This trend is consistent with Strunnikova et al.’s results in which they reported the transcriptome changes in response to stress after HQ stimulation [35].

### Effects of replicative senescence

To examine the risk factor of aging, we used multiple, serial passage as in vitro model of aging for RPE cells. While some may consider that the method of serial passage may not mimic aging macular RPE cells in vivo, serial passage remains one of the most common methods for modeling aging of many cell types in vitro. High passage RPE possesses important aging characteristics such as decreased proliferation capacity and increased senescence-associated β-galactosidase activity [10,14,15]. β-galactosidase activity was associated with the increased lysosomal enzymes observed in senescent cells and was found in aged in vivo RPE cells [36,37]. Our data demonstrated that cell death increased in late passage RPE culture (P21) compared to early passage RPE (P5; Figure 3), which is consistent with the observation that RPE cell density decreases with increasing age in normal human eyes [7]. The increased cell death rate in late passage RPE cells might be explained by our gene expression data published earlier [13]. The continuous telomere and telomeric DNA loss in late passage RPE cells may compromise the capacity of RPE cells to maintain the original survival rate. This is consistent with the observed downregulation of heat shock protein, αB-crystallin, known to be a prosurvival factor [12].

**Figure 7:**
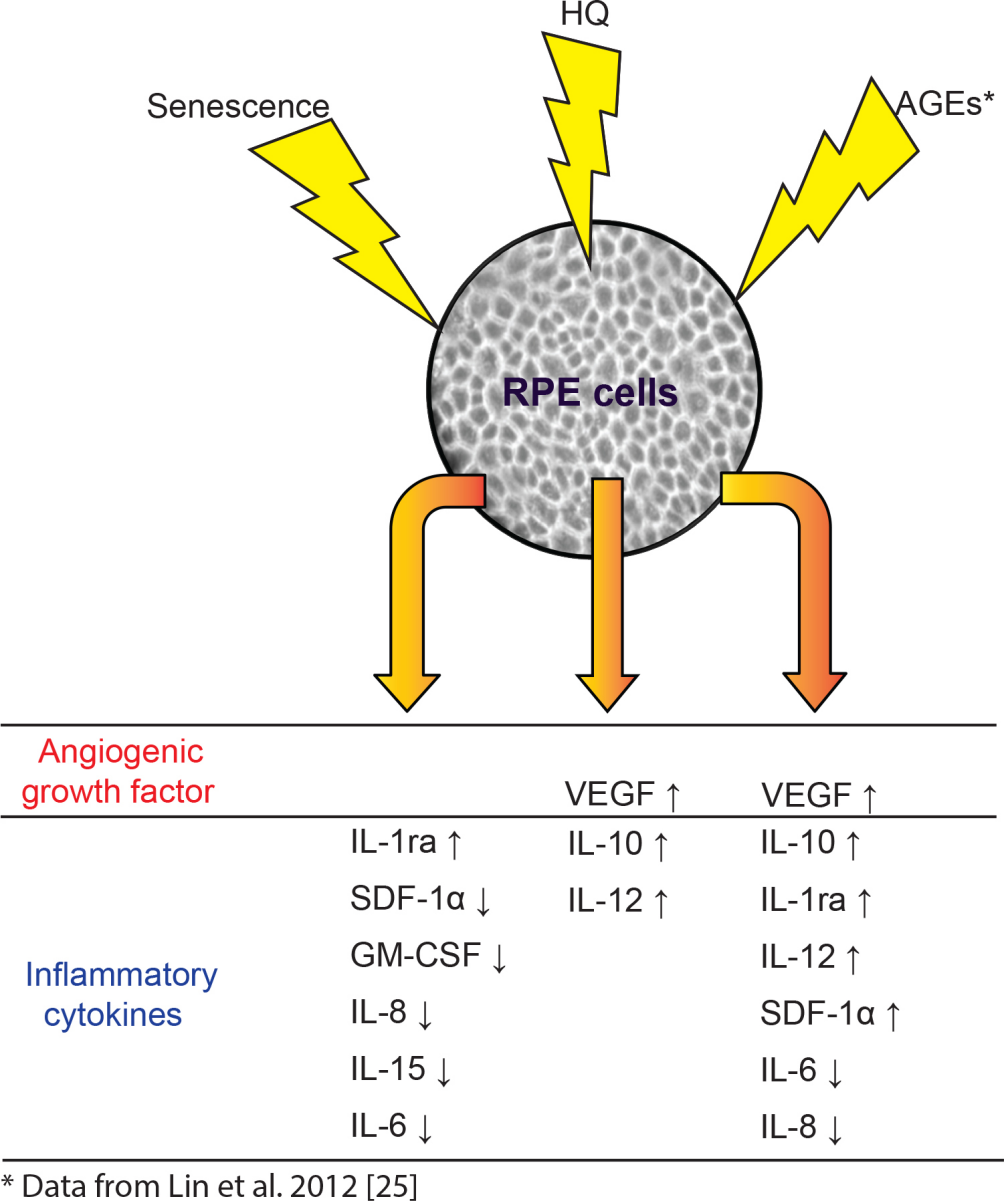
Summary schematic of secretion profiles from RPE under different stimulations: HQ, passage- related senescence, and AGEs. Secreted profiles included various cytokines/growth factors involved in angiogenesis and inflammation. The change is indicated by arrows: an up arrow for an increase and a down arrow for a decrease.

In terms of the secretion profile of late passage RPE cells, five cytokines/growth factors related to proinflammatory were downregulated, including IL-6, IL-15, IL-8, GM-CSF, and SDF-1α. The inability to produce proinflammatory cytokines in senescent RPE cells was also found in one previous gene expression study [17]. Interestingly, this inability is likely cell-type specific, as Shelton et al. showed that the senescent fibroblasts and senescent vascular endothelial cells demonstrated more robust proinflammatory gene expression than senescent RPE [13,17]. Meanwhile, the overexpression of anti-inflammatory IL-1ra was found in late passage RPE cells as well as in post-mortem donor eyes from older eyes (≥70 years old). Since IL-1β expression is increased in older eyes [38], the senescent RPE cells in normal physiologic conditions might develop a protective mechanism against the imbalance of proinflammatory and anti-inflammatory factors due to stimuli such as oxidative stress and drusen (Figure 7) [39]. This will require further studies.

The secretion profile of late passage RPE cells in the current study showed a generally reduced proinflammatory trend, while in the literature other senescent cells types such as fibroblasts tend to secrete many proinflammatory cytokines [40,41]. The difference might come from the unique characteristics intrinsic to the RPE cell type as other studies demonstrated that senescent RPE cells had a less robust proinflammatory profile than senescent fibroblasts [17,42]. In addition, our in vitro results demonstrate a different trend compared to in vivo studies [43,44]. This difference is likely due to the complexity of multiple cell types and their interactions in ocular tissues in vivo models in contrast to in vitro culture work in which only one cell type is studied.

In conclusion, the current study focuses on the secretion profile of RPE cells under aging and HQ-induced oxidative stress, two well-known risk factors for AMD. Our data suggest that under HQ-induced oxidative stress and replicative senescence, RPE cells secrete cytokines and growth factors that maintain homeostasis in the outer retina and prevent further imbalances of proinflammatory and anti-inflammatory factors. The ability of RPE cells to respond in a specific pattern to known risk factors of AMD, such as oxidative stress and age-associated changes, in vitro further supports their key role in the pathogenesis of AMD (Figure 7).

## Appendix 1. Changes of tested molecules in RPE treated with HQ.

To access the data, click or select the words “Appendix 1.”

## Appendix 2. Changes of tested molecules in P21 and P5 RPE.

To access the data, click or select the words “Appendix 2.”
